# The COVID-19 outbreak: The issue of face masks

**DOI:** 10.1017/ice.2020.129

**Published:** 2020-04-13

**Authors:** Ming-Wei Wang, Yong-ran Cheng, Lan Ye, Meng-Yun Zhou, Juan Chen, Zhan-hui Feng

**Affiliations:** 1 Department of Cardiology, Affiliated Hospital of Hangzhou Normal University, Hangzhou, China; 2 Zhejiang Academy of Medical Sciences, Hangzhou, China; 3 Hangzhou Medical College, Hangzhou, China; 4 Basic Medical College, Guizhou Medical University, Guizhou, China; 5 Department of Neurology, Affiliated Hospital of Guizhou Medical University, Guiyang, China

*To the Editor—*We appreciate the report by Wang et al^1^ regarding the role of masks and respirators in protecting against the SARS-Cov-2 virus. However, with the progression of the COVID-19 epidemic, by April 1, 2020, 75,948 cases had been confirmed by the World Health Organization, and 36,571 deaths from the outbreak had been declared. Face masks can prevent human-to-human aerosol transmission of such infections effectively^2,3^; thus, Asian countries such as China, South Korea, and Japan encourage the public to wear face masks in public areas.

China has adopted its own country-specific comprehensive prevention and control measures, such as closing communities and cities, banning parties, delaying school, and restricting work, requiring face masks in public areas, etc. The domestic COVID-19 epidemic was effectively controlled in China in mid-March 2020. Requiring face masks played an important role in this success, and it should not be abandoned.^4,5^

**Fig. 1:**
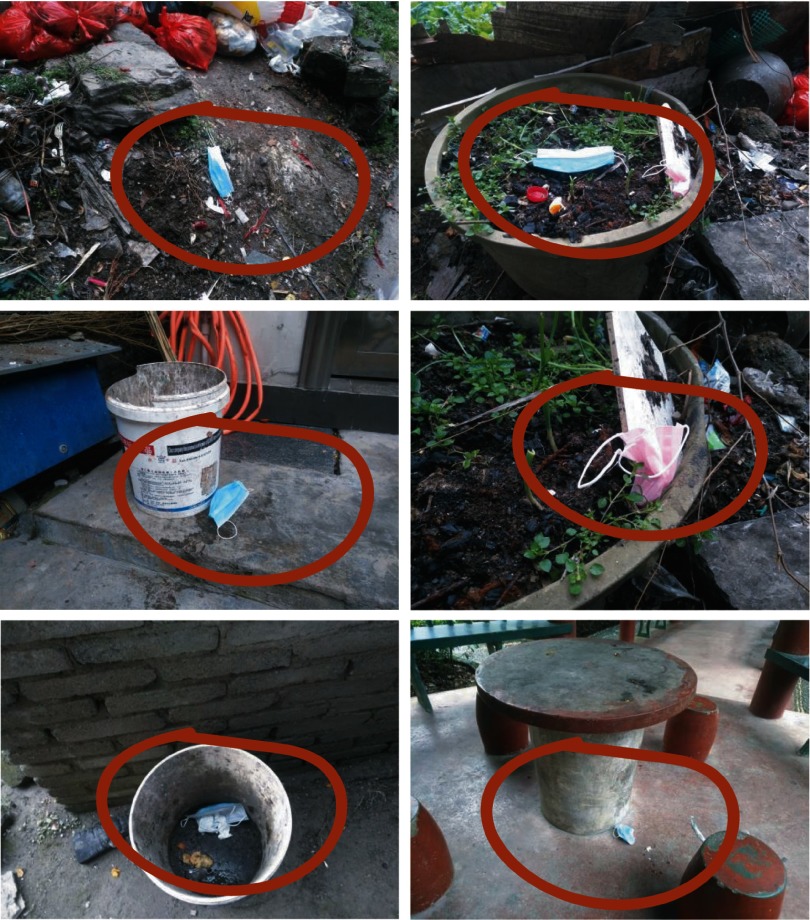
Discarded face masks

As more people began to wear face masks with the SARS-Cov-2 outbreak, face masks began to become scarce at the beginning of 2020. Now it has become difficult for the public to buy face masks. In addition, face masks become ineffective and must be disposed of. Discarded face masks have been found in many places, such as the street, parks, buses, hospitals, train stations, etc (Fig. 1).

We think that 3 measures should be undertaken: (1) improve the supply of masks; (2) promote public awareness about how to deal with discarded masks; (3) carry out innovation to improve masks. Ultimately, we believe that we will conquer SARS-Cov-2 outbreak.
